# The effects of dual antiplatelet therapy (DAPT) adherence on survival in patients undergoing revascularization and the determinants of DAPT adherence

**DOI:** 10.1186/s12872-022-02677-8

**Published:** 2022-05-23

**Authors:** Shuqi Zhang, Mithlesh Chourase, Nupur Sharma, Sujata Saunik, Mona Duggal, Goodarz Danaei, Bhanu Duggal

**Affiliations:** 1 Department of Epidemiology, Harvard TH Chan School of Public Health, Boston, USA; 2 Health Technology Assessment Hub, AIIMS Rishikesh, Rishikesh, India; 3 Government of Maharashtra, Mumbai, India; 4 Department of Community Medicine, PGIMER, Chandigarh, India; 5 Department of Global Health and Population, Harvard TH Chan School of Public Health, Boston, USA; 6 Department of Cardiology, AIIMS Rishikesh, Rishikesh, India

**Keywords:** Dual antiplatelet therapy (DAPT), Percutaneous coronary intervention (PCI), Coronary artery disease, Adherence, India

## Abstract

**Background:**

The prevalence and burden of coronary heart disease (CHD) has increased substantially in India, accompanied with increasing need for percutaneous coronary interventions (PCI). Although a large government-funded insurance scheme in Maharashtra, India covered the cost of PCI for low-income patients, the high cost of post-PCI treatment, especially Dual Antiplatelet Therapy (DAPT), still caused many patients to prematurely discontinue the secondary prevention. Our study aimed to investigate the effectiveness of DAPT adherence on all-cause mortality among post-PCI patients and explore the potential determinants of DAPT adherence in India.

**Method:**

We collected clinical data of 4,595 patients undergoing PCI in 110 participating medical centers in Maharashtra, India from 2012 to 2015 by electronic medical records. We surveyed 2527 adult patients who were under the insurance scheme by telephone interview, usually between 6 to 12 months after their revascularization. Patients reporting DAPT continuation in the telephone survey were categorized as DAPT adherence. The outcome of the interest was all-cause mortality within 1 year after the index procedure. Multivariate Cox proportional hazard (PH) model with adjustment of potential confounders and standardization were used to explore the effects of DAPT adherence on all-cause mortality. We further used a multivariate logistic model to investigate the potential determinants of DAPT adherence.

**Results:**

Out of the 2527 patients interviewed, 2064 patients were included in the analysis, of whom 470 (22.8%) discontinued DAPT prematurely within a year. After adjustment for baseline confounders, DAPT adherence was associated with lower one-year all-cause mortality compared to premature discontinuation (less than 6-month), with an adjusted hazard ratio (HR) of 0.52 (95% Confidence Interval (CI) = (0.36, 0.67)). We also found younger patients (OR per year was 0.99 (0.97, 1.00)) and male (vs. female, OR of 1.30 (0.99, 1.70)) had higher adherence to DAPT at one year as did patients taking antihypertensive medications (vs. non medication, OR of 1.57 (1.25, 1.95)).

**Conclusion:**

These findings suggest the protective effects of DAPT adherence on 1-year mortality among post-PCI patients in a low-income setting and indicate younger age, male sex and use of other preventive treatments were predictors of higher DAPT adherence.

**Supplementary Information:**

The online version contains supplementary material available at 10.1186/s12872-022-02677-8.

## Background

Cardiovascular disease (CVD) is a leading cause of mortality and morbidity in India. According to Global Burden of Disease (GBD) study 2019, the estimated number of deaths caused by CVD was 2.57 million, constituting 27.4% of all-cause mortality and resulting in 13.9% of Disability-Adjusted Life Years (DALYs) lost in India [1]. As one of the main cardiovascular diseases, coronary heart disease (CHD) has increasing prevalence and mortality in both urban and rural population due to aging and epidemics of diabetes, hypertension, and dyslipidemia [2, 3]. Percutaneous coronary intervention (PCI) with implantation of bare-metal stents (BMSs) or drug-eluting stents (DESs) is an effective treatment for CHD patients. Compared with conventional medical management, PCI reduces risk of death, cardiac death, and myocardial infraction (MI) in patients with unstable coronary artery disease, although the benefit over medical therapy in stable coronary artery disease (SCAD) is still uncertain [4]. To date, there have been several international guidelines [5, 6], providing comprehensive guidance for revascularization in CHD patients. According to these guideline, patients undergoing PCI generally should receive Dual Antiplatelet Therapy (DAPT), a combination of aspirin and oral antiplatelet agents for at least 6 months to decrease the risk of death and recurrent events [6].

Coronary interventions in India continue to increase year by year [7]. In 2012, to make the health services more available for the poor, the government of Maharashtra launched a government-funded insurance scheme, covering 971 medical procedures including PCI under designated hospitals [8, 9]. Under the insurance scheme, the number of annual coronary interventions doubled from 2013 to 2017 in Maharashtra, India [10]. However, the insurance scheme does not cover post-PCI treatment including DAPT, so patients from lower socioeconomic backgrounds may discontinue treatment prematurely due to high cost or low health literacy [11]. The ‘real-life’ effectiveness of DAPT on patients’ all-cause mortality could differ due to non-adherence in India, compared with previous populations examined in high- or middle-income settings [12–14].

Despite global consensus on the management of CHD, gaps in the adherence to DAPT as secondary prevention still exist in developing countries [15]. There were several studies describing the implementation of guideline-directed medical therapy for patients with acute coronary syndrome (ACS) in India [16, 17], but few studies investigated the effects and predictors of DAPT adherence. To fill this gap, we examined the effect of DAPT adherence on all-cause mortality within one year after patients underwent PCI and separately explored the determinants of DAPT adherence in Maharashtra, India.

## Methods

### Study sites and population

This prospective study included 110 medical centers in each district of Maharashtra. Participating centers had adequate facilities to provide standardized cardiovascular care and patients covered under the government-funded insurance scheme were free to present to any of the participating centers to receive treatment [8, 9]. For this study, we used patients with CAD whose procedures conducted between 2012 and 2015 and followed them between August 2012 and November 2016. Eligible study participants were adult patients (aged 18 years and above) receiving PCI in one or more coronary arteries at a participating center under the government-funded insurance scheme. The choice of stent and post-PCI medications was at the discretion of the cardiologist. Patients who did not receive a stent during the index PCI and patients who died during hospitalization for the index procedure were excluded. We further excluded patients who had missing values in exposure, outcomes, and potential confounders.

### Data collection

We used data from two sources: (1) complete electronic medical records of the treated patients and procedural details, maintained by the Department of Health and Family Welfare of the Government of Maharashtra; (2) telephone interviews with the patients usually conducted between 6 and 12 months after the index PCI by the research interviewers to collect information on adherence to DAPT, survival and other covariates. If people died before 6 months after PCI, the next of the kin would be interviewed using the same questionnaire (Additional file 1: Material S1). Prior to the start of the study, the research coordinators responsible for data collection participated in a training session where the standardized forms for data collection and manual of operations were reviewed to ensure consistency in data collection practices. Permission to obtain only verbal informed consent over the telephone (rather than written consent) was granted by the Ethics Committee.

### Definitions

We assumed all the patients started to use DAPT directly after PCI as the guidelines recommend. Patients reporting current DAPT use (both antiplatelet and aspirin use) in the telephone survey were categorized as DAPT adherence. Since most interviews were conducted between 6 to 12 months after PCI, the adherence duration was supposed to be more than 6 months. For patients who died before 6 months, we categorized them as adherence if they used DAPT when they were still alive. The outcome of the study was all-cause mortality within 1 year after the index procedure. The specific date of death was from the telephone survey reported by the next of the kin. Supplementary material 2 provides information on how the variables in our study were measured.

### Statistical analysis

We first used appropriate statistics (t-test or X^2^-test) to compare patients’ demographic characteristics, clinical characteristics, socioeconomic characteristics, lifestyle, revascularization details, medicine use and self-reported drug availability by DAPT adherence and non-adherence.

Then, we used a Log-rank test to compare the survival difference between the DAPT adherent and non-adherent patients. We further used a multivariate Cox proportional hazard (PH) model to analyze the effects of DAPT adherence on one-year survival after PCI. We chose a set of potential confounders based on a-priori knowledge about common causes of adherence and mortality, including age, sex, hypertension, diabetes, eligible CAD type, status of tobacco use, education level, employment status, revascularization in Mumbai or not, sent location, stent type, year of PCI, total number of stents, total stent length. We tested the proportional hazard assumption using Schoenfeld residuals [18]. We then used the estimated coefficients, baseline hazard and the joint distribution of baseline confounders in the entire participants to estimate standardized Kaplan–Meier survivor curves by adherence status [19]. We also conducted subgroup analysis by age, sex, CAD type and antihypertensive treatment and test the effect modification using likelihood ratio test. For sensitivity analysis, we excluded patients who died before 6 months after procedure, then analyzed the effect of DAPT adherence to test the robustness of our study results.

Finally, A multivariate logistic regression model was used to explore the potential determinants of adherence to DAPT based on available information and the recommended analysis of factors reported to affect medicine adherence by American College of Preventive Medicine (ACPM) [20]. Potential determinants in the analysis included age, sex, hypertension, diabetes, eligible CAD type, status of tobacco use, education level, employment status, revascularization in Mumbai or not, year of PCI, drug availability, drug affordability and closeness to pharmacy. Statistical analysis was performed using the R studio version 1.4.

## Results

**Fig. 1:**
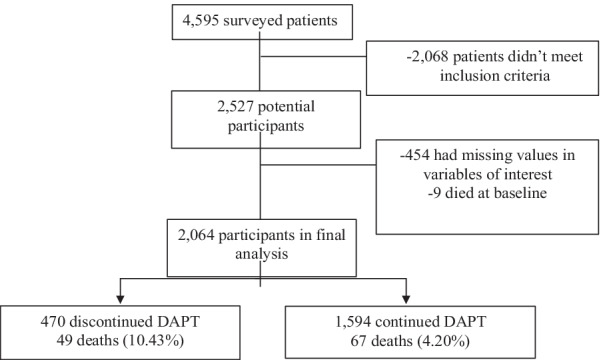
Selection process for participants based on inclusion and exclusion criteria

Out of 4595 patients (or family members in case of deceased patients) interviewed between August 2012 and November 2016, 2527 adult patients underwent PCI under the insurance scheme and had interview after their PCI. We further excluded 454 patients who had missing values in exposure, time of death, and potential confounders, and 9 patients who died at the hospital after the index PCI (Fig. 1). Of the 2,064 patients included in final analysis, 470 (22.8%) discontinued DAPT prematurely within a year.

Mean age was 56.71 years old (SD = 10.77) and 75.6% of eligible patients were men. As shown in Table 1, the DAPT adherent and non-adherent population were remarkably similar with minor differences in baseline characteristics. Specifically, the proportion of men in DAPT adherent group was higher than DAPT non-adherent group (76.5% vs. 72.3%), and the DAPT adherent group were slightly younger (56.38 years old (SD = 10.77) vs. 57.85 years old (SD = 10.72)). 91.2% of DAPT-adherent patients used Clopidogrel and Aspirin, 10.1% used Prasugrel and Aspirin, and 1.3% used Ticlopidine and Aspirin. DAPT adherent group were more likely to take antihypertensive medications (65.1% vs. 56.2%). DAPT-adherent patients were more likely to report that the prescribed drugs are easily available at the pharmacy (73.8% vs. 70.2%), they were able to afford the medicine (33.8% vs. 29.8%) and they lived close to a pharmacy/dispensary (64.3% vs. 57.7%).

**Table 1:** Patient’s characteristics, PCI details separately by DAPT adherence and non-adherence (N = 2019)

Variables	Total	DAPT non-adherence (n = 470)		DAPT adherence (n = 1594)		*P* value
		N	%	N	%	
Age (years)^a^	2064	57.85 (mean)	10.72 (SD)	56.38 (mean)	10.77 (SD)	0.01
Age < 60 years old	1124	233	49.6%	891	55.9%	0.02
Age ≥ 60 years old	940	237	50.4%	703	44.1%	
Sex						0.06
Female	504	130	27.7%	374	23.5%	
Male	1560	340	72.3%	1220	76.5%	
Hypertension	830	195	41.5%	635	39.8%	0.56
Diabetes	662	157	33.4%	505	31.7%	0.52
Eligible CAD type						0.44
Previous MI	289	73	15.5%	216	13.6%	
Acute coronary syndrome	1126	246	52.3%	880	55.2%	
Chronic stable angina/positive stress test	649	151	32.1%	498	31.2%	
Status of tobacco use						0.18
Non-smoker	1511	356	75.7%	1155	72.5%	
Current/past smoker	553	114	24.3%	439	27.5%	
Education in middle school or above	1059	240	51.1%	819	51.4%	0.95
Employed	864	197	41.9%	667	41.8%	1.00
Underwent intervention in Mumbai	676	138	29.4%	538	33.8%	0.08
Stent locations						0.26
LAD	854	198	42.1%	656	41.2%	
RCA	388	75	16.0%	313	19.6%	
LCX	195	51	10.9%	144	9.0%	
Multiple stents	627	146	31.1%	481	30.2%	
Stent type						0.27
BMS	1057	256	54.5%	801	50.3%	
DES	934	198	42.1%	736	46.2%	
DES and BMS	73	16	3.4%	57	3.6%	
Year of PTCA						0.29
2012	441	99	21.1%	342	21.5%	
2013	718	150	31.9%	568	35.6%	
2014	413	95	20.2%	318	19.9%	
2015	492	126	26.8%	366	23.0%	
Stent details		mean	SD	mean	SD	
Total number of stents per patient^a^		1.52 (mean)	0.70 (SD)	1.52 (mean)	0.66 (SD)	0.89
Total stent length per patient (mm)^a^		30.75 (mean)	19.48 (SD)	31.18 (mean)	19.61 (SD)	0.67
Medicine use						
Aspirin	1767	173	36.8%	1594	100.0%	< 0.001
Clopidogrel	1544	90	19.1%	1454	91.2%	< 0.001
Prasugrel	182	21	4.5%	161	10.1%	< 0.001
Ticlopidine	22	2	0.4%	20	1.3%	0.20
Antihypertension	1301	264	56.2%	1037	65.1%	< 0.001
Self-reported drug availability						
Drugs easily available	1507	330	70.2%	1177	73.8%	0.13
Medicine affordable	678	140	29.8%	538	33.8%	0.12
Close to pharmacy	1296	271	57.7%	1025	64.3%	0.01

MI, myocardial infraction; BMS, bare-metal stent; DES, drug-eluting stent; LAD, left anterior descending artery; LCX, left circumflex artery; RCA, right coronary artery; PTCA, Percutaneous Transluminal Coronary Angioplasty

^a^Continuous variable, mean and standard deviation are presented

**Fig. 2:**
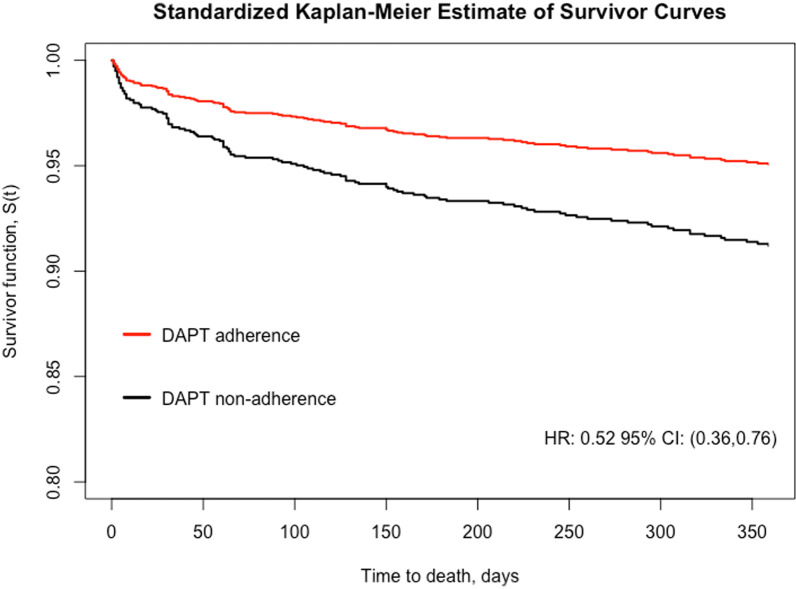
Standardized Kaplan Meier estimate of one-year all-cause mortality for DAPT adherence versus non-adherence

We observed 116 deaths within 1 year of the index PCI procedure, of whom 91 died before 6 months after PCI. The crude death rate in DAPT adherent patients was 4.2% compared with 10.4% among DAPT non-adherent patients (*P* < 0.001 in log-rank test). Figure 2 showed the standardized cumulative rates of post-PCI mortality over the course of one year by DAPT adherence and non-adherence.

The adjusted hazard ratio (HR) for 1-year all-cause mortality of DAPT adherence was 0.52 with 95% Confidence Interval (CI) = (0.36, 0.76) (Table 2). The tests for Schoenfeld residuals of DAPT treatment and all covariates in the model were not statistically significant, so the assumptions of proportional hazards held.

**Table 2:** Hazard ratios (HR) and 95% confidence interval for one-year all-cause mortality of DAPT adherence estimated from multivariate Cox proportional hazard model (N = 2019)

Variables	Multivariate cox PH model		
	HR	95%CI	*P* value
DAPT adherence versus non-adherence	0.52	(0.36, 0.76)	< 0.001
Age (years)	1.05	(1.03, 1.07)	< 0.001
Male versus female	0.91	(0.56, 1.47)	0.69
Hypertension versus no hypertension	1.03	(0.68, 1.54)	0.90
Antihypertensive treatment versus no treatment	0.08	(0.05, 0.14)	< 0.001
Diabetes versus no diabetes	1.43	(0.97, 2.1)	0.07
Eligible CAD type			
Chronic stable angina/positive stress test	Ref	Ref	Ref
Previous myocardial infraction	1.56	(0.89, 2.71)	0.12
Acute coronary syndrome	0.99	(0.65, 1.51)	0.96
No smoking versus smoking	1.09	(0.71, 1.67)	0.71
Underwent PCI out of versus in Mumbai	0.71	(0.45, 1.13)	0.15
Middle school and above versus none or up to primary school	1.36	(0.91, 2.03)	0.14
Employed versus unemployed	1.12	(0.73, 1.72)	0.61
Stent location			
LAD	Ref	Ref	Ref
RCA	0.43	(0.22, 0.83)	0.01
LCX	0.42	(0.16, 1.05)	0.06
Multiple locations	0.75	(0.43, 1.33)	0.33
Stent type			
BMS	Ref	Ref	Ref
DES	0.97	(0.64, 1.45)	0.86
DES and BMS	0.47	(0.14, 1.55)	0.21
Year of PTCA			
2012	Ref	Ref	Ref
2013	0.92	(0.53, 1.6)	0.77
2014	1.66	(0.88, 3.12)	0.12
2015	2.25	(1.20, 4.23)	0.01
Total length of stents	1.01	(1.00, 1.03)	0.02
Total number of stents (mm)	1.04	(0.68, 1.59)	0.85

BMS, bare-metal stent; DES, drug-eluting stent; LAD, left anterior descending artery; LCX, left circumflex artery; RCA, right coronary artery; PTCA, percutaneous transluminal coronary angioplasty

Reference group: Female; no diabetes; no hypertension; not having antihypertensive treatment; chronic stable angina/positive stress test; current/past smoker; underwent intervention in Mumbai; none or up to primary school; not employed; only stent on LAD; only stent with BMS; PTCA in 2012

Model adjusted for age, sex, hypertension, diabetes, eligible CAD type, status of tobacco use, education level, employment status, revascularization in Mumbai or not, sent location, stent type, year of PCI, total number of stents, total stent length

We found no significant interaction between age and CAD types and DAPT adherence, but the survival effects of DAPT adherence seemed stronger among female and patients not taking antihypertensive drug (Table 3). However, the number of events among patients with antihypertensive treatment was too small for a meaningful comparison of the survival benefit by antihypertensive treatment. In our sensitivity analysis (Table 4), after excluding patients who died before 6 months, DAPT adherence was found not associated with all-cause mortality (HR of 0.62 (0.27, 1.44)).

**Table 3:** Hazard ratios (HR) and 95% confidence interval for one-year all-cause mortality of DAPT adherence in the subgroup analysis and test of effect modification

Variables	Multivariate Cox PH model		
	HR	95% CI	*P* value
Age categories			
< 60 years old	0.50	(0.26, 0.99)	0.99
≥ 60 years old	0.52	(0.32, 0.84)	
Sex			
Male	0.66	(0.42, 1.04)	0.07
Female	0.24	(0.11, 0.56)	
Eligible CAD type			
Previous myocardial infraction	0.32	(0.11, 0.97)	0.41
Acute coronary syndrome	0.49	(0.29, 0.85)	
Chronic stable angina/positive stress test	0.78	(0.37, 1.62)	
Antihypertensive treatment			
Yes	1.92	(0.43, 8.64)	0.06
No	0.44	(0.29, 0.66)	

Model adjusted for the same covariates as the primary multivariate Cox proportional hazard model except for the corresponding stratified variables. *P* value were estimated using likelihood ratio test of the interaction term

**Table 4:** Hazard ratios (HR) and 95% confidence interval for one-year all-cause mortality of DAPT adherence in sensitivity analysis

Variables	Multivariate Cox PH model		
	HR	95%CI	*P* value
As reported adherence or non-adherence	0.52	(0.36, 0.76)	< 0.001
Excluding deaths before 6 months	0.62	(0.27, 1.44)	0.27

Model adjusted for the same covariates as the primary multivariate Cox proportional hazard model, including age, sex, hypertension, diabetes, eligible CAD type, status of tobacco use, education level, employment status, revascularization in Mumbai or not, sent location, stent type, year of PCI, total number of stents, total stent length

Among the potential determinants of adherence to DAPT examined in the model, only younger age (OR per year was 0.99 (0.97, 1.00)), male (vs. female, OR of 1.30 (0.99, 1.70)) and taking antihypertensive medication (vs. non medication, OR of 1.57 (1.25, 1.95)) were important determinants of higher adherence (Table 5).

**Table 5:** Association (odds ratios OR and 95% confidence intervals) between baseline characteristics and DAPT adherence at 6–12 months (N = 2064)

Variables	Multivariate logistic model		
	OR	95%CI	*P* value
Age (years)	0.99	(0.97, 1.00)	0.01
Male versus female	1.30	(0.99, 1.7)	0.06
Hypertension versus no hypertension	0.86	(0.69, 1.08)	0.20
Antihypertensive treatment versus no treatment	1.59	(1.27, 1.99)	0.00
Diabetes versus no diabetes	0.96	(0.76, 1.21)	0.73
Eligibility event type			
Chronic stable angina/positive stress test	Ref	Ref	Ref
Previous myocardial infraction	0.89	(0.64, 1.25)	0.49
Acute coronary syndrome	1.15	(0.91, 1.46)	0.25
No smoking versus smoking	1.15	(0.9, 1.48)	0.27
Underwent PCI out of versus in Mumbai	0.97	(0.75, 1.25)	0.81
Middle school and above versus none or up to primary school	0.86	(0.68, 1.08)	0.19
Employed versus not employed	0.82	(0.64, 1.06)	0.13
Year of PTCA			
2012	Ref	Ref	Ref
2013	1.12	(0.83, 1.5)	0.45
2014	1.04	(0.74, 1.47)	0.81
2015	0.84	(0.6, 1.18)	0.31
Drugs unavailability versus availability	0.99	(0.76, 1.3)	0.96
Drug unaffordability versus affordability	0.93	(0.72, 1.19)	0.55
Not close to versus close to pharmacy	0.81	(0.62, 1.04)	0.10

Reference group: female; no diabetes; no hypertension; not taking anti-HTN; chronic stable angina/positive stress test; current/past smoker; in Mumbai; none or up to primary school; not employed; PTCA in 2012; drugs available; medicine affordable; close to pharmacy

## Discussion

Our results indicated the protective effects of DAPT adherence after PCI on one-year all-cause mortality among a low-income population in India. The US and European guidelines recommend DAPT after PCI for at least 6 months in stable coronary artery disease and for at least 12 months in ACS [5, 21]. In our study population, 86.0% of patients had either ACS or stable SCAD, but only 77.2% reported DAPT adherence after PCI. Even among the relatively higher DAPT adherence groups, the proportions of adherence were still below 80% (men: 78.2%, young patients: 79.3%, having antihypertensive treatment: 79.7%). We further examined potential determinants of DAPT adherence: in Indian settings, men, younger patients, and those taking antihypertension drug had higher DAPT adherence. However, we couldn’t find other determinants that affects DAPT adherence significantly.

DAPT is among the most intensively investigated treatment options in the field of cardiovascular medicine [21]. As secondary prevention, DAPT adherence plays an important role in improving individuals’ clinical outcomes and achieving cost-effectiveness of medical interventions [22, 23]. Our study findings are consistent with many previous observational studies that also indicated the survival benefits of longer-term DAPT adherence. For instance, based on the BIFURCAT registry, a study found that extended DAPT (> 12 months) was associated with a lower incidence of Major Adverse Cardiac Events (MACE) compared with intermediate-term DAPT (6–12 months) driven by a reduction of all-cause death in the acute coronary syndrome cohort [24]. A prior study also found that the four-year mortality for patients with coronary bifurcation lesion was significantly lower when accepting more than 12-month DAPT after PCI than accepting less than12-month DAPT [25]. Furthermore, the PARIS study found that patients who ceased DAPT due to brief interruption (for surgery) or disruption (non-compliance or because of bleeding) had significant higher hazard of MACE compared to people who remained on DAPT [26]. In subgroup analysis, we didn’t find significant difference in the protective effects of DAPT on survival by sex, which was same as the results of a sex-specific patient-level pooled-analysis of randomized trials [27].

The American College of Preventive Medicine has proposed 5 key groups of factors that affect adherence: (1) socioeconomic factors, (2) health care system–related factors, (3) medical condition– related factors, (4) therapy-related factors and (5) patient-related factors [20]. Our results on higher adherence among younger patients, males and those on antihypertensive drugs is consistent with previous investigations of adherence in similar populations [28, 29]. However, similar to other electronic medical record systems, we did not have access to data on several dimensions of adherence as proposed by ACPM, such as socioeconomic factors and patient-related factors such as health literacy, knowledge about DAPT and confidence in dealing with symptoms caused by DAPT [11]. Furthermore, we found only a weak association between self-reported measures of access to and affordability of healthcare and adherence. Therefore, further studies are needed to elucidate the determinants of DAPT adherence.

There are several strengths of our study. Firstly, responding to the increasing prevalence of CHD [30] and the emerging need for PCI [10], we examined the current status of DAPT adherence as secondary prevention and its effects on mortality in India using electronic medical records for the entire state of Maharashtra. Secondly, our findings contribute to the knowledge of potential determinants of DAPT adherence in real-world studies. Specifically, the study targeting Indian population helps address knowledge gap in resource-poor settings, as most previous studies were conducted in high-income populations, like the US and European countries [26, 28, 29]. However, our study has several limitations. Firstly, the self-reported DAPT adherence in telephone survey might be over-estimated and cause misclassification. If patients tended to report DAPT-adherence even when they didn’t, the non‐differential misclassification of exposure would lead the results toward the null value of no association. Secondly, we did not have information on the exact dose and duration of DAPT use by individuals, therefore we had to rely on self-reported DAPT use from the telephone survey, rather than more accurate measurement tools [31]. Thirdly, drug availability and affordability were self-reported variables measured by simple questions, and their validity was untested. In addition, we didn’t have information on potential socioeconomic confounders, such as income, work type, and health literacy as well as information on kidney function, and drug safety, such as bleeding. Due to potentially unadjusted confounding, our findings should be interpreted cautiously.

Our results have important implications for clinical practice and public health research. To improve post-PCI survival, more efforts should be taken to improve patients’ DAPT adherence, while taking individual’s clinical presentation and bleeding risk into accounts [32]. Additionally, future studies including qualitative and mixed methods studies are required to provide more evidence on the determinants of DAPT adherence, especially regarding socioeconomic and health system-related factors.

## Conclusion

To conclude, using prospective data from electronic medical records and telephone interview of patients undergoing PCI, we found that DAPT adherence was significantly associated with lower risk of one-year all-cause mortality. Additionally, younger patients, men, and those on antihypertensive treatment had higher DAPT adherence. Future studies, including qualitative and mixed methods studies should be conducted to further investigate the determinants of adherence in this population.

## Supplementary Information

**Additional file 1. **The Better Health Heart Survey.

## Data Availability

The questionnaire used for data collection can be found in supplementary material. Since the dataset used by the study includes patients’ identities and private information, it is only available from the corresponding author on reasonable request.

## Abbreviations

CVD: Cardiovascular disease
CHD: Coronary heart disease
ACS: Acute coronary syndrome
SCAD: Stable coronary artery disease
MI: Myocardial infraction
GBD: Global Burden of Disease
DALYs: Disability-adjusted life years
PCI: Percutaneous coronary intervention
BMSs: Bare-metal stents
DESs: Drug-eluting stents
DAPT: Dual Antiplatelet Therapy
MACE: Major Adverse Cardiac Events
ACPM: American College of Preventive Medicine
LAD: Left anterior descending artery
LCA: Left circumflex artery
RCA: Right coronary artery
PH: Proportional hazard
HR: Hazard ratio
CI: Confidence interval

## References

[1] Adler AI, Stratton IM, Neil HA, Yudkin JS, Matthews DR, Cull CA, Wright AD, Turner RC, Holman RR (2000). Association of systolic blood pressure with macrovascular and microvascular complications of type 2 diabetes (UKPDS 36): prospective observational study. BMJ.

[2] Gupta R, Guptha S, Sharma KK, Gupta A, Deedwania P (2012). Regional variations in cardiovascular risk factors in India: India heart watch. World J Cardiol.

[3] Gupta R, Mohan I, Narula J (2016). Trends in coronary heart disease epidemiology in India. Ann Glob Health.

[4] Chacko L, Rajkumar C, Nowbar AN, Kane C, Mahdi D, Foley M, Shun-Shin M, Cole G, Sen S (2020). Effects of percutaneous coronary intervention on death and myocardial infarction stratified by stable and unstable coronary artery disease: a meta-analysis of randomized controlled trials. Circ Cardiovasc Qual Outcomes.

[5] Levine GN, Bates ER, Bittl JA, Brindis RG, Fihn SD, Fleisher LA, Granger CB, Lange RA, Mack MJ, Mauri L (2016). 2016 ACC/AHA guideline focused update on duration of dual antiplatelet therapy in patients with coronary artery disease: a report of the American College of Cardiology/American Heart Association task force on clinical practice guidelines. J Am Coll Cardiol.

[6] Neumann FJ, Sousa-Uva M, Ahlsson A, Alfonso F, Banning AP, Benedetto U, Byrne RA, Collet JP, Falk V, Head SJ (2019). 2018 ESC/EACTS guidelines on myocardial revascularization. Eur Heart J.

[7] Ramakrishnan S, Mishra S, Chakraborty R, Chandra KS, Mardikar HM (2013). The report on the Indian coronary intervention data for the year 2011–National Interventional Council. Indian Heart J.

[8] Duggal B, Gokul B, Duggal M, Saunik S, Singh P, Agrawal A, Singh K, Wadhera P, Anupindi R, Nallamothu BK (2019). Drug-eluting stent use among low-income patients in maharashtra after statewide price reductions. Circ Cardiovasc Interv.

[9] Duggal B, Subramanian J, Duggal M, Singh P, Rajivlochan M, Saunik S, Desiraju K, Avhad A, Ram U, Sen S (2018). Survival outcomes post percutaneous coronary intervention: Why the hype about stent type? Lessons from a healthcare system in India. PLoS ONE.

[10] Arramraju SK, Koganti S, Janapati R, Emmareddy SK, Mandala GR (2019). The report on the Indian coronary intervention data for the year 2017-National Interventional Council. Indian Heart J.

[11] Pithara C, Pufulete M, Johnson TW, Redwood S (2020). Patient perspectives of nuisance bleeding and adherence to dual antiplatelet therapy: a qualitative study. Open Heart.

[12] Trial of invasive versus medical therapy in elderly patients with chronic symptomatic coronary-artery disease (TIME): a randomised trial. The Lancet. 2001; 358(9286):951–7. 10.1016/S0140-6736(01)06100-1 11583747

[13] De Bruyne B, Pijls NH, Kalesan B, Barbato E, Tonino PA, Piroth Z, Jagic N, Mobius-Winkler S, Rioufol G, Witt N (2012). Fractional flow reserve-guided PCI versus medical therapy in stable coronary disease. N Engl J Med.

[14] Kolandaivelu K, Leiden BB, O'Gara PT, Bhatt DL (2014). Non-adherence to cardiovascular medications. Eur Heart J.

[15] Guha S, Sethi R, Ray S, Bahl VK, Shanmugasundaram S, Kerkar P, Ramakrishnan S, Yadav R, Chaudhary G, Kapoor A (2017). Cardiological Society of India: position statement for the management of ST elevation myocardial infarction in India. Indian Heart J.

[16] Sawhney JPS, Mullasari A, Kahali D, Mehta V, Nair T, Kaul U, Hirematth MS (2019). Short- and long-term follow-up of antithrombotic management patterns in patients hospitalized with acute coronary syndrome: Indian subgroup of EPICOR Asia study. Indian Heart J.

[17] George NE, Shukkoor AA, Joseph N, Palanimuthu R, Kaliappan T, Gopalan R (2022). Implementation of clinical audit to improve adherence to guideline-recommended therapy in acute coronary syndrome. Egypt Heart J.

[18] Schoenfeld D (1982). Partial residuals for the proportional hazards regression model. Biometrika.

[19] Danaei G, Garcia Rodriguez LA, Cantero OF, Logan RW, Hernan MA (2018). Electronic medical records can be used to emulate target trials of sustained treatment strategies. J Clin Epidemiol.

[20] Abu SM, Banu A, Khan AR, Hussain MZ (1995). Prevalence of diabetes and hypertension in a rural population of Bangladesh. Diabetes Care.

[21] Valgimigli M, Bueno H, Byrne RA, Collet JP, Costa F, Jeppsson A, Juni P, Kastrati A, Kolh P, Mauri L (2018). 2017 ESC focused update on dual antiplatelet therapy in coronary artery disease developed in collaboration with EACTS: The Task Force for dual antiplatelet therapy in coronary artery disease of the European Society of Cardiology (ESC) and of the European Association for Cardio-Thoracic Surgery (EACTS). Eur Heart J.

[22] Bansilal S, Castellano JM, Garrido E, Wei HG, Freeman A, Spettell C, Garcia-Alonso F, Lizano I, Arnold RJ, Rajda J (2016). Assessing the impact of medication adherence on long-term cardiovascular outcomes. J Am Coll Cardiol.

[23] Chowdhury R, Khan H, Heydon E, Shroufi A, Fahimi S, Moore C, Stricker B, Mendis S, Hofman A, Mant J (2013). Adherence to cardiovascular therapy: a meta-analysis of prevalence and clinical consequences. Eur Heart J.

[24] Filippo O, Kang J, Bruno F, Han JK, Saglietto A, Yang HM, Patti G, Park KW, Parma R, Kim HS (2021). Benefit of extended dual antiplatelet therapy duration in acute coronary syndrome patients treated with drug eluting stents for coronary bifurcation lesions (from the BIFURCAT Registry). Am J Cardiol.

[25] Jang WJ, Ahn SG, Song YB, Choi SH, Chun WJ, Oh JH, Cho SW, Kim BS, Yoon JH, Koo BK (2018). Benefit of prolonged dual antiplatelet therapy after implantation of drug-eluting stent for coronary bifurcation lesions: results from the coronary bifurcation stenting registry II. Circ Cardiovasc Interv.

[26] Mehran R, Baber U, Steg PG, Ariti C, Weisz G, Witzenbichler B, Henry TD, Kini AS, Stuckey T, Cohen DJ (2013). Cessation of dual antiplatelet treatment and cardiac events after percutaneous coronary intervention (PARIS): 2 year results from a prospective observational study. The Lancet.

[27] Sawaya FJ, Morice MC, Spaziano M, Mehran R, Didier R, Roy A, Valgimigli M, Kim HS, Woo Park K, Hong MK (2017). Short-versus long-term dual antiplatelet therapy after drug-eluting stent implantation in women versus men: a sex-specific patient-level pooled-analysis of six randomized trials. Catheter Cardiovasc Interv.

[28] Malik J, Yousaf H, Abbasi W, Hameed N, Mohsin M, Shahid AW, Fatima M (2021). Incidence, predictors, and outcomes of DAPT non-compliance in planned vs ad hoc PCI in chronic coronary syndrome. PLoS One.

[29] Moalem K, Baber U, Chandrasekhar J, Claessen BE, Sartori S, Aquino M, Dangas G, Iakovou I, Colombo A, Kini A (2019). Incidence, predictors, and outcomes of DAPT disruption due to non-compliance vs bleeding after PCI: insights from the PARIS Registry. Clin Res Cardiol.

[30] Gupta R, Joshi P, Mohan V, Reddy KS, Yusuf S (2008). Epidemiology and causation of coronary heart disease and stroke in India. Heart.

[31] Peterson AM, Nau DP, Cramer JA, Benner J, Gwadry-Sridhar F, Nichol M (2007). A checklist for medication compliance and persistence studies using retrospective databases. Value Health.

[32] Costa F, Vranckx P, Leonardi S, Moscarella E, Ando G, Calabro P, Oreto G, Zijlstra F, Valgimigli M (2015). Impact of clinical presentation on ischaemic and bleeding outcomes in patients receiving 6- or 24-month duration of dual-antiplatelet therapy after stent implantation: a pre-specified analysis from the PRODIGY (Prolonging Dual-Antiplatelet Treatment After Grading Stent-Induced Intimal Hyperplasia) trial. Eur Heart J.

