# Supplementary material for: The effects of dual antiplatelet therapy (DAPT) adherence on survival in patients undergoing revascularization and the determinants of DAPT adherence
Source: BMC Cardiovasc Disord. 2022 May 23;22:238. doi: 10.1186/s12872-022-02677-8 (PMC9125829; doi:10.1186/s12872-022-02677-8)
Supplement: Supplementary file 1 — Additional file 1. The Better Health Heart Survey. [file 12872_2022_2677_MOESM1_ESM.docx]

**Supplementary material 1**

***Better Health Heart Survey***

***Instructions***

▪ To be administered by the Research Interviewer

▪ Informed consent must be obtained prior to administering this questionnaire. Signed consent form to be stored in the Master file.

Subject ID ……………….. **[** | | **]**

Age (in completed years):

Age: Years **[** | **]**

Sex: 01 Female, 02 Male **[** | **]**

Date: DD/MM/YY

Address: House Number

Gali no.

Village

District

Date of PTCA

Hospital where PTCA done **[** | | | | | **]**

Site ID **[** | **]**

Who is answering the questions

Self/Relation to patient

| Informed Consent obtained | 01Yes, 02 No | **[** \| **]** |
| --- | --- | --- |
| Date of informed consent | DD/MM/YY | **[** \| \| \| \| \| **]** |

Telephone number (self) -----------------------

Alternative phone number

When available? -------------------------

Research Interviewer ……………………………………… **[** | **]**

Introduction:

I am ………………………….. I will be interviewing you today and will be asking you some questions about your health and background. Your answers will be kept completely confidential and will not be linked to your name or personal information. May I continue? Thank you.

In case the person you are supposed to ask the questions has died and you are able to talk to the next of the kin please ask their permission and ask questions in the questionnaire.

Questions answered by ________self 01

Questions answered by _______next of kin 02

If 02 Please specify____________________(open ended)

Start time [ / ]

| **Screening Questions (Please proceed if the respondent answers yes to these questions.)** | | | | |
| --- | --- | --- | --- | --- |
| **SECTION 2 Socio Demographic questionnaire** | | | | |
| 101 | What is your highest educational level? | Illiterate(01) Literate without formal education(02) Below primary(03) Primary(04) Middle(05) Secondary/ Class-10/ Matric(06) Hr. Sec/ Sec/ Class XII/ Pre-Univ(07) Graduate and above(08))  Vocational **09**  Other(88 | **[** \| **]** |  |
| 102 | What is your current marital status? | Never married(1) Married(2) Remarried(3) Widow(er)(4) Divorced(5) Seperated(6) Refused to anwser(98) | **[** \| **]** |  |
| 103 | Who do you currently live with at home?  **(Multiple responses possible. After respondent answers, probe by asking for any others)**  **Do NOT read out answers.** | a) By your self ……………………  b) spouse …………………  c) Parents …………………………  d) Siblings ………………………..  e) Children ……………………….  f) Relatives ……………………….  g) Friends / Roommates ………….  **N/A 98** | **[** \| **]**  **[** \| **]**  **[** \| **]**  **[** \| **]**  **[** \| **]**  **[** \| **]**  **[** \| **]** |  |
| 104 | Are You employed | Yes **01**  No **02** | **[** \| **]** |  |
| 105 | If yes do you work on full time or part time basis? | full time **01**  part time basis **02** | **[** \| **]** |  |
| 105 | If no, why don’t you work? | Don’t want to work **01**  Lost job **02**  Too sick to carry on working **03**  Don’t need to work **04**  Never worked **05**  Other (specify) **88**  **N/A 98** |  |  |

| 106 | Are the following facilities available at your  home?  **Read out and code all that apply**  a) Land ………………………………  b) House ……………………………..  c)Car ………………………………..  d) TV …………………………………..  e) Motorbike / scooter ………………….  f) Refrigerator ………………………..  j) AC ………………………………  k) Computer / printer …….……………  l) Washing machine ………………….  m) Air cooler ……….…………  n) Cycle ………………..…………... | Yes **01** ; No **02 ;** N/A **98;** DNK **99** | | | |  |
| --- | --- | --- | --- | --- | --- | --- |
|  |  |  | Do you own  the following? | Was it  purchased last year? | Can you sell it  if necessary? |  |
|  |  | **[** \| **] [** \| **] [** \| **]**  **[** \| **] [** \| **] [** \| **]**  **[** \| **] [** \| **] [** \| **]**  **[** \| **] [** \| **] [** \| **]**  **[** \| **] [** \| **] [** \| **]**  **[** \| **] [** \| **] [** \| **]**  **[** \| **] [** \| **] [** \| **]**  **[** \| **] [** \| **] [** \| **]**  **[** \| **] [** \| **] [** \| **]**  **[** \| **] [** \| **] [** \| **]** | | | |  |

|  | **SECTION 2: Risk factors for cardiovascular disease** | | | | | | | | | | |
| --- | --- | --- | --- | --- | --- | --- | --- | --- | --- | --- | --- |
| 201 | Do you have Diabetes | | 01 yes  02 No  Do not Know -99  (DNK) | | |  | | | ------------------- | | |
| 202 | If yes when did you have your last blood sugar level done? ( days) | |  | | | **[** \| **]** | | |  | | |
| 203 | What was your last fasting blood sugar level | | Do not remember-00 | | | **[** \| \| **]** | | |  | | |
| 204 | What was your last postprandial blood sugar level | | Do not remember-00 | | | **[** \| \| **]** | | |  | | |
| 205 | What was your HbA1c level | | Do not remember-00 | | | **[** \| \| **]** | | |  | | |
| 206 | Do you have Blood pressure(hypertension) | | 01 yes  02 No  Do not Know -99 | | | | | | **[** \| **]** | | |
| 207 | If yes, for how many years | |  | | | | | |  | | |
| 207 | What was your last BP | | Systolic/diastolic  Example : 120 /80 | | | Systolic [  ]  Diastolic [  ]  Do not remember-00  If yes | | |  | | |
| 208 | How often have you had an alcoholic  drink in the last 30 days (one month)? | | Never **01**  Once **02**  2-3 times in a month? **03**  Once or twice a week **04**  3-4 times a week **05**  Nearly every day **06**  Daily **07** | | | **[**  **]** | | |  | | |
| 209 | Are you a Smoker | | 01 Yes  02 No | | | | [  ]  If yes | |  | |  |
| 210 | Which of the following best describes your tobacco smoking status? | | Current smoker  (last 6 months) 01 Past smoker  (6 months ago) 02  Non-smoker 03 | | | | [  ] | |  | |  |
| 211 | What do you smoke?  Multiple answers are possible | | Cigarrette 01  Bidi 02  Chew tobacco 03 | |  ]   ]   ] | | | If yes please ask the frequency   \| If current smoker, frequency- beedis per day \| \| --- \| \| If current smoker, frequency- cigarettes per day \| \| If current smoker, frequency- others per day \| | 01 Once a month  02 2-3 times a month  03 Once or twice a week  04 3-4 times a week  05 Nearly every day  06 Daily  98 N/A | | [  ]  [  ]  [  ] |
| 212 | Any other substance abuse  (Please specify and go to next in case of multiple drugs) | 01 Yes  02 No | | If yes------------ | | | | 01 Once a month  02 2-3 times a month  03 Once or twice a week  04 3-4 times a week  05 Nearly every day  06 Daily  98 N/A | | [  ] | |
| 213 | Family History of CAD | 01 yes  02 No | |  | | | | 01 Mother  Age at which she had CAD  02 Father  Age at which she had CAD  03 Sister  Age at which she had CAD  04  Brother  Age at which she had CAD  05  Additional siblings with CAD | | ------------ | |
| 214 | History of Sudden death in the family | 01 Yes  02 No | |  | | | |  | |  | |

|  | **SECTION 3: CURRENT USE of Cardiovascular Drugs** | | |  |  |
| --- | --- | --- | --- | --- | --- |
| 301 | How many drugs are you taking?  Please could you tell us which you are currently taking, the doses and frequency?  ***Interviewer***  (1) To keep samples of all medications being used currently at the health facility to facilitate recall.  (2) To examine medication wrapping from patient | | | |  |
|  |  |  |  |  |  |
| **302** | **Drug Regimen**  **Antiplatelet agents Dose Frequency** | | | | |
| a)  b)  c)  d) | Aspirin Clopidogrel  Ticlopidine  Prasugrel | Yes/No mg | Once a day /twice a day 0 | | **01/02**  **01/02**  **01/02**  **01/02** |
| 303 | Antihypertension drugs  Atenolol/others specify  Others | | | | |
| 304 | Antidiabetic drugs  Oral/Insulin | | | | |

| **Availability of Drugs** | |  |  |  |
| --- | --- | --- | --- | --- |
| 305 | Prescribed drugs are easily available at the pharmacy | Yes **01**  No **02** | [ \| ] | If yes please answer q 509  If no please skip to q 510 |
| 306 | If yes, are you able to afford the required medicine | Yes **01**  No **02** | [ \| ] |  |
| 307 | Is the pharmacy/ dispensary close to your home  If yes please answer question 511 | Yes **01**  No **02** | [ \| ] |  |
| 308 | Would you like the government to have designated pharmacy centers where you can pick up the medicine | Yes **01**  No **02** | [ \| ] |  |
| 309 | Would you like to be informed through sms or an automated call when the drugs become available | Yes **01**  No **02** | [ \| ] |  |
| 310 | Would you like to be able to send an SMS or call or when your supply is finishing | Yes **01**  No **02** | [ \| ] |  |

| **SECTION 4. SIDE EFFECTS - BASED ON PATIENT REPORTED SYMPTOMS** | | | |
| --- | --- | --- | --- |
|  | We would like you to answer some questions about the kind of symptoms and  feelings you have been having. Your answers will help us understand the effects of the medication you are taking.  The following questions ask about the symptoms you might have had during the  **past four weeks (30 days or month)**  **0. I do not have this symptom**  **1. It doesn’t bother me**  **1. It bothers me a little**  2. **It bothers me a lot**  **Read out and circle the correct answer**  **Use visual scale to indicate degree of severity** | |  |
| 401 | Dyspepsia, stomach pain (grade mild, moderate, severe), stomach cramps | 0 1 2 3 | \| **]** |
| 402 | Feeling dizzy or lightheaded/ headaches | 0 1 2 3 | \| **]** |
| 403 | Nausea or vomiting/ diarrhoea | 0 1 2 3 | \| **]** |
| 404 | Difficulty in breathing, chest tightness | 0 1 2 3 | \| **]** |
| 405 | Difficulty falling or staying asleep | 0 1 2 3 | \| **]** |
| 406 | Skn rashes/deep purple or blue bruises,bleeding while brushing, nosebleed,  Black stools, blood in urine or stools | 0 1 2 3 | \| **]** |
| 407 | Seizures, depression, anxiety | 0 1 2 3 | \| **]** |
| 408 | Difficulty in micturition | 0 1 2 3 | \| **]** |
| 409 | Arthralgias/back pain | 0 1 2 3 | \| **]** |

| **SECTION 5 : Follow up** | | | |  | | | |
| --- | --- | --- | --- | --- | --- | --- | --- |
| 501 | Have you had a Lipid profile done? | Yes **01**  No **02**  Don’t know **99** | | [ \| ] | | |  |
| 502 | Have you had chest pain after the procedure? | Yes **01**  No **02**  Don’t know **99** | | [ \| ] | |  | |
| 503 | Have you ever been hospitalized again after your PTCA ? | Yes **01**  No **02**  Don’t know **99** | | [ \| ] | |  | |
| 504 | Have you undergone a repeat procedure? The previous stent was blocked or there was a new block in a different vessel? | Same vessel01  Different vessel 02  Don’t know99 | | [ \| ] | |  | |
| 505 | Please tell me how many times have  you been hospitalized for cardiovascular related illnesses over in the past 12 months? | Total number of hospitalizations  N/A **98** | | [ \| ] | |  | |
| **Please ask the questions in case the patient has died** | | | | | | | |
| 506 | When did the patient die |  | [ \| ] | | Open ended  Please put months if less than a month say less than a month | | |
| 507 | After how many months of surgery did the patient die |  | [ \| ] | | Please put months if less than a month say less than a month | | |
| 508 | Where did the patient die | Home 01  On way to hospital 02  Transferring from one hospital to other 03  At Hospital 04 | [ \| ] | |  | | |
| 509 | Why do you feel the patient died  **(Multiple responses possible. After respondent answers, probe by asking for any others.)** | Lack of medicines01  Lack of medical facilities nearby02  Lack of emergency services 03  Any other 04 | [ \| ]  [ \| ]  [ \| ]  [ \| ] | | Multiple answers possible | | |

| **SECTION 6 : BELIEFS AND PERCEPTIONS REGARDING medicines for cardiovascular THERAPY** | | | |
| --- | --- | --- | --- |
| **These statements are designed to learn more about your thoughts and views about medications. There are no right or wrong answers here. Please feel free to tell us what you think. How strongly do you agree or disagree with the following statements.** | | | |
| 601 | Medications are necessary once a stent has been deployed? | **Yes 01**  **No 02**  **Do not know 99** | [ \| ] |
| 602 | Medications should be stopped only after consultation with a cardiologist/any doctor | Yes 01  No 02  Don’t know 99 | [ \| ] |
| 603 | Disease has become less serious because of  Medications | Strongly disagree **01**  Disagree **02**  Agree **03**  Strongly agree **04** | [ \| ] |
| 604 | Medications are not worth taking because  of serious side effects | Strongly disagree **01**  Disagree **02**  Agree **03**  Strongly agree **04** | [ \| ] |
| 605 | Within a few months after PTCA  Surgery , medications can be stopped | Strongly disagree **01**  Disagree **02**  Agree **03**  Strongly agree **04** | [ \| ] |

| **SECTION 7 : PHQ2 Screening for Depression** | | | |
| --- | --- | --- | --- |
| **These statements are designed to screen for depression.** | | | |
| 701 | Little interest or pleasure in doing things | Not at all 01  Several days 02  More than half the days 03  Nearly every day 04 | [ \| ] |
| 702 | Feeling depressed , down or hopeless | Not at all 01  Several days 02  More than half the days 03  Nearly every day 04 | [ \| ] |
| **SECTION 8 : Questions on Internet/Mobile Phones** | | | |
| **These statements are designed to learn more about your thoughts and views about mobile phones and internet** | | | |
| 801 | What would you prefer call from a nurse, automated reminder, both or neither as a reminder for taking medicine If no why?? | Yes 01  No 02  Don’t know 99 | [ \| ] |
| 802 | Would you like to have a helpline that would it help you to report any adverse events or other emergencies | Yes 01  No 02  Don’t know 99 | [ \| ] |
| 803 | Are you likely to report an adverse event more if you have mobile airtime as an incentive to report this event | Yes 01  No 02  Don’t know 99 | [ \| ] |
| 804 | Do you use internet on phone or at home?/  Email address | Yes 01  No 02  Don’t know 99 | [ \| ] |
| 805 | Do you have an email account? | Yes 01  No 02  Don’t know 99 | [ \| ] |

| **SECTION 9: SOCIAL SUPPORT** | | | | | | | | |  |
| --- | --- | --- | --- | --- | --- | --- | --- | --- | --- |
| 901 | | Do you receive support from your family? | | Yes **01**  No **02** | | **[** \| **]** | |  |  |
| 902 | | What kind of support do you get from  your family?  **(Multiple responses possible. After respondent answers, probe by asking for any others)**  **Do NOT read out answers.** | | Mentioned **01**  Not Mentioned **02**  N/A **98**  a) Emotional / psychological support …… b) Financial support …………………….. c) Physical care and support ……………..  d) Other (specify) | | **[** \| **] [** \| **] [** \| **] [** \| **]** | |  |  |
| 903 | | In general, how satisfied are you with the  overall support (help) you get from your family? | | Not satisfied **01**  Not quite satisfied **02**  Moderately satisfied **03**  Very satisfied **04**  **N/A 98** | | **[** \| **]** | |  |  |
| 904 | | Do you receive support from outside your  home or family members? | | Yes **01**  No **02** | | **[** \| **]** | |  |  |
| 905 | From where do you get support?  **(Multiple responses possible. After respondent answers, probe by asking for any others)**  **Do NOT read out answers.** | | Mentioned **01**  Not Mentioned **02**  N/A **98**  a) Friends ……………………………….  b) NGO ………………………………… c) Community based organization ……… d) Religious based organization ……….. e) Government facility ………………….. f) Workplace programs …………………  g) Other (specify) | | **[** \| **] [** \| **] [** \| **] [** \| **] [** \| **] [** \| **] [** \| **]** | |  | | |
| 906 | What kind of support do you get fromoutside your home?  **(Multiple responses possible. After respondent answers, probe by asking for any others)**  **Do NOT read out answers.** | | Mentioned **01**  Not Mentioned **02**  N/A **98**  a) Psychological support …………  b) Financial support ……………… c) Physical care and support …….. d) Income generating activities ….. e) Nutritional support ……………. f) Legal support …………………..  g) Other (specify) | | **[** \| **] [** \| **] [** \| **]**  **[** \| **]**  **[** \| **] [** \| **]**  **[** \| **]** | |  | | |
| 007 | In general, how satisfied are you with the overall support you get from your outside your home? | | Not satisfied **01**  Not quite satisfied **02**  Moderately satisfied **03**  Very satisfied **04**  **N/A 98** | | **[** \| **]** | |  | | |

**End time [----------------]**

**Thank you for participating.**

**Supplementary material 2**

**Measurement of variables used in the analysis**

| **Variable** | **Description** | **Values** | **Data collection** |
| --- | --- | --- | --- |
| **Time-related variables** | | |  |
| Date_of_PTCA | The date of Percutaneous transluminal coronary angioplasty | DATE | Electronic medical records (in analysis) & Telephone interview (for validity) |
| Dt_Interview | The date of interview | DATE | Telephone interview |
| DT_death | The date of death | DATE | Telephone interview |
| **Outcome-related variables** | | | Telephone interview |
| Is_Dead | Status of death | 1 = No  2 = Yes | Telephone interview |
| **Demographic characteristics** | | |  |
| Age_tmnt | Age at enrollment | Continuous | Electronic medical records (in analysis) |
| Age_followup | Age through interview | Continuous | Telephone interview (for validity) |
| Sex_tmnt | Sex | 0=Female  1=Male | Electronic medical records (in analysis) |
| Sex_followup | Sex through interview | 1=Female  2=Male | Telephone interview (for validity) |
| **Clinical characteristics** | | |  |
| CAD History | Entry events | 0=Missing information  1=Previous MI  2=Acute coronary syndrome  3=Chronic stable angina  4=Positive stress test | Electronic medical records |
| Diabetes | Reported diabetes | 0 = No  1 = Yes | Telephone interview |
| Hypertension | Reported hypertension | 0 = No  1 = Yes | Telephone interview |
| CAD_family history | Family History of Coronary artery disease | 0 = No  1 = Yes | Telephone interview |
| **Lifestyle variables** | | |  |
| Tobacco_status | Status of tobacco use | 0 = N/A  1 = Current smoker (last 6 months)  2 = Past smoker (6 months ago)  3 = Non-smoker | Telephone interview |
| Alcohol_cat | Status of drinking | 0 = No  1 = Yes | Telephone interview |
| SES variables | | |  |
| Site_ID | Location of hospital | 0 = Mumbai  1 = Out of Mumbai | Telephone interview |
| Education_level | Level of education | 0 = None_Little (None or up to primary school)  1 = Some_Higher (Middle school and above) | Telephone interview |
| Employed_status | Employment status | 0 = No  1 = Yes | Telephone interview |
| **Characteristics of PCI stent** | | |  |
| year_ptca | Year of PTCA | Year (2012-2015) | Electronic medical records |
| LAD_Sent | PCI stent for left anterior descending | 0=BMS (bare mental stent)  1=DES (drug-eluting stent) | Electronic medical records |
| LCX_Sent | PCI stent for left circumflex artery | 0=BMS (bare mental stent)  1=DES (drug-eluting stent) | Electronic medical records |
| RCA_Sent | PCI stent for right coronary artery | 0=BMS (bare mental stent)  1=DES (drug-eluting stent) | Electronic medical records |
| LAD_Number_of_Sent | Number of PCI stents for left anterior descending | Continuous | Electronic medical records |
| LCx_Number_of_Sent | Number of PCI stents for left circumflex artery | Continuous | Electronic medical records |
| RCA_Number_of_Sent | Number of PCI stents for right coronary artery | Continuous | Electronic medical records |
| LAD_ Sent_Width | Width of PCI stents for left anterior descending | Continuous | Electronic medical records |
| LAD_Distal_Sent_Width | Width of PCI distal stents for left anterior descending | Continuous | Electronic medical records |
| LAD_Proximal_Sent_Width | Width of PCI proximal stents for left anterior descending | Continuous | Electronic medical records |
| LAD_Wideth_Additional_Stent | Width of PCI additional stent for left anterior descending | Continuous | Electronic medical records |
| LAD_ Sent_Length | Length of PCI stents for left anterior descending | Continuous | Electronic medical records |
| LAD_Distal_Sent_Length | Length of PCI distal stents for left anterior descending | Continuous | Electronic medical records |
| LAD_Proximal_Sent_Length | Length of PCI proximal stents for left anterior descending | Continuous | Electronic medical records |
| LAD_Length_Additional_Stent | Length of PCI additional stent for left anterior descending | Continuous | Electronic medical records |
| LCx_Sent_Width | Width of PCI stents for left circumflex artery | Continuous | Electronic medical records |
| LCx_Distal_Sent_Width | Width of PCI distal stents for left circumflex artery | Continuous | Electronic medical records |
| LCx_Proximal_Sent_Width | Width of PCI proximal stents for l left circumflex artery | Continuous | Electronic medical records |
| LCx_Additional_Sent_Width | Width of PCI additional stent for left circumflex artery | Continuous | Electronic medical records |
| LCx_ Sent_Length | Length of PCI stents for left circumflex artery | Continuous | Electronic medical records |
| LCx_Distal_Sent_Length | Length of PCI distal stents for left circumflex artery | Continuous | Electronic medical records |
| LCx_Proximal_Sent_Length | Length of PCI proximal stents for left circumflex artery | Continuous | Electronic medical records |
| LCx_Length_Additional_Stent | Length of PCI additional stent for left circumflex artery | Continuous | Electronic medical records |
| RCA_Sent_Width | Width of PCI stents for right coronary artery | Continuous | Electronic medical records |
| RCA_Distal_Sent_Width | Width of PCI distal stents for right coronary artery | Continuous | Electronic medical records |
| RCA_Proximal_Sent_Width | Width of PCI proximal stents for l right coronary artery | Continuous | Electronic medical records |
| RCA_Additional_Sent_Width | Width of PCI additional stent for right coronary artery | Continuous | Electronic medical records |
| RCA_ Sent_Length | Length of PCI stents for right coronary artery | Continuous | Electronic medical records |
| RCA_Distal_Sent_Length | Length of PCI distal stents for right coronary artery | Continuous | Electronic medical records |
| RCA_Proximal_Sent_Length | Length of PCI proximal stents for right coronary artery | Continuous | Electronic medical records |
| RCA_Length_Additional_Stent | Length of PCI additional stent for right coronary artery | Continuous | Electronic medical records |
| stent.type | PCI stent types | 0=BMS (bare mental stent)  1=DES (drug-eluting stent) | Electronic medical records |
| lad_stent_present | Indicator for stents on left anterior descending | 0 = No  1 = Yes | Electronic medical records |
| lcx_stent_present | Indicator for stents on left circumflex artery | 0 = No  1 = Yes | Electronic medical records |
| rca_stent_present | Indicator for stent on right coronary artery | 0 = No  1 = Yes | Electronic medical records |
| total_number_of_stents | Total number of stents per patient | Continues | Electronic medical records |
| max_max_width | Maximum stent diameter | Continues | Electronic medical records |
| min_min_width | Minimum stent diameter | Continues | Electronic medical records |
| total_length | Total stent length per patient | Continues | Electronic medical records |
| **Medicine use** | | |  |
| Aspirin | Use of any aspirin or aspirin type pills | 0 = No  1 = Yes | Telephone interview |
| Clopidogrel | Use of Clopidogrel | 0 = No  1 = Yes | Telephone interview |
| Prasugrel | Use of Prasugrel | 0 = No  1 = Yes | Telephone interview |
| Ticlopidine | Use of Ticlopidine | 0 = No  1 = Yes | Telephone interview |
| Dapt | Adherence of DAPT between 6 and 12 months | 0 = No  1 = Yes (Aspirin with one or more of Clopidogrel/ Prasugrel/Ticlopidine) | Telephone interview |
| Antihypertension | Taking Antihypertension drugs or not | 1 = Yes  2 = No | Telephone interview |
| **Availability of Drugs** | | |  |
| Drugs_availibility | Prescribed drugs are easily available at the pharmacy | 1 = Yes  2 = No | Telephone interview |
| Medicine_afford | If yes, are you able to afford the required medicine | 1 = Yes  2 = No | Telephone interview |
| Pharmacy_close | Is the pharmacy/ dispensaryclose to your home? | 1 = Yes  2 = No | Telephone interview |
